# Effect of cementation techniques on fracture load of monolithic zirconia crowns

**DOI:** 10.1080/26415275.2021.1990764

**Published:** 2021-10-20

**Authors:** Janne Angen Indergård, Anneli Skjold, Christian Schriwer, Marit Øilo

**Affiliations:** Faculty of Medicine, Department of Clinical Dentistry, University of Bergen, Bergen, Norway

**Keywords:** Ceramic dental crown, luting agent, zirconium dioxide

## Abstract

**Aim:**

The aim of this study was to evaluate the effect of cement on the fracture load of monolithic zirconia crowns with different yttria content (3 and 5 mol%).

**Methods:**

A total of 62 monolithic zirconia crowns, 40 3Y-zirconia crowns (Prettau^®^ Zirconia, Zirkonzahn) and 22 5Y-zirconia crowns (Prettau^®^ 4 Anterior^®^, Zirkonzahn) were produced to a shallow chamfer molar preparation. The 3Y-crowns were divided into four groups and attached to composite abutment duplicates (SDR^®^ flow+, Dentsply DeTrey GmbH) using the following four cementation techniques; (1) Self-adhesive resin-based cement, (2) Pre-treatment with air-abrasion and self-adhesive resin-based cement, (3) Zinc phosphate cement, (4) Glass-ionomer cement. The 5Y-crowns were divided into two groups and attached to the duplicates with; (1) Self-adhesive resin-based cement, or (2) Air-abrasion pre-treatment and self-adhesive resin-based cement. All crowns were loaded axially (0.5 mm/min) on the occlusal surface until fracture occurred.

**Results:**

Among the 3Y-zirconia groups, the zinc phosphate cement group fractured at lower loads compared to the resin-based cement groups, with and without air-abrasion, (*p* < .012). Among the 5Y-groups the air-abraded crowns fractured at statistically significant lower loads compared to the untreated crowns (*p* < .028). Load at fracture values were significantly different between the two zirconia materials (*p* < .001), with fracture loads ranging from 3873 to 7500 N in the 3Y-groups, and 2100 to 4948 N in the 5Y-groups.

**Conclusions:**

Resin-based cementation increased the fracture load compared to non-adhesive cementation. The 3Y-crowns fractured at almost twice the loads of the 5Y-crowns. Pre-treatment with air abrasion reduced the strength of the 5Y-crowns only, showing the importance of differentiating the treatment of the two materials.

## Background

The development of yttria-stabilized tetragonal zirconia polycrystalline (Y-TZP) for dental purposes has resulted in a range of different products which can be used as a core material as well as a monolithic restoration without the use of veneering porcelain [1,2]. The material group possesses several beneficial qualities such as high fracture toughness and strength, as well as good biocompatibility [3,4]. There are several aspects that can explain the good mechanical properties of zirconia. The industrial production of homogeneous zirconia blocks leads to few flaws and imperfections [5]. The machining of restorations results in restorations with good fit and fewer flaws than hand-made restorations [5]. There are several different types of dental zirconia materials available. The main difference among the materials is the amount of added stabilizing oxide, ranging from 3 mol% (1st generation) up to >5mol% (3rd generation) [2,3]. A yttria content of 3 mol% results in the metastable 3Y-TZP (3Y zirconia). Increasing the amount of yttria to 5 mol% results in a partially stabilized zirconia with a high cubic content (5Y-PSZ, 5Y zirconia). The increase leads to an enhancement in the materials’ optical properties, as cubic phase crystals are transparent [6]. The increased content of the cubic phase has, however, shown to give a significant reduction in the mechanical properties of the materials [3,7,8].

The traditional 3Y-TZP material possesses stress-induced transformation toughening capabilities. Zirconia crystals stabilized in a tetragonal crystal structure can transform from the tetragonal [*t*] to monoclinic [*m*] phase when exposed to stress. The small volumetric increase following this transition causes compressive stress around the crack front, making it less likely to propagate [9]. With a higher amount of yttria, a larger number of crystals will be fully stabilized in the cubic phase and thus a t-m transformation is less likely to happen [3,7]. This may negatively affect the clinical survival time for the 5Y zirconia compared to the 3Y zirconia, but the evidence is lacking.

Retention loss and fractures are still the main technical complications registered in clinical trials of zirconia-based restorations [10]. The type of cement used, and cementation technique can potentially affect both crown strength and retention. It is uncertain which cement is optimal as there are some contradictory results on the effect of types of cement on fracture load in previous studies [11–13]. Furthermore, there is limited evidence regarding the best method of pre-treating the zirconia surfaces for optimal bonding [12,14,15]. Some studies have shown that enhanced adhesion can be achieved through mechanical and chemical surface treatments such as airborne particle abrasion (air-abrasion), tribochemical silica coating, or the use of phosphate groups in the bonding or cement [12,14–17].

Conventional types of cement such as zinc-phosphate and glass ionomer cement are efficient as retention is based on micromechanical interlocking between the tooth surface and the inner walls of the restoration [18]. Furthermore, opaque cement will reduce the overall appearance of an all-ceramic restoration. Adhesive cementation technique with the additional benefit of chemical adhesion between tooth and restoration is on the other hand time-consuming and technique sensitive.

Most adhesion studies conducted are on 3Y zirconia, while less information is available for 5Y zirconia [11,12,19]. Additionally, few clinical studies address the question of the effect of cementation technique on high-strength ceramic crowns, and little difference is found [19]. The exception is for glass-ceramic and silicate-based ceramic restorations which both shows increased survival rates with adhesive compared to conventional cementation technique [19]. Clinically, there are several variables that can affect the loss of retention and fracture rates, which makes clinical trials impractical for addressing the current question without a large number of participants [11]. Well-designed *in vitro* tests can give better insight into the material’s behavior *in vivo*.

The aim of this study was to evaluate the effect of different types of cement and cementation techniques on the load at fracture and fracture mode in monolithic zirconia crowns with two different material compositions.

The null hypothesis tested was that type of cement and cementation technique does not affect fracture load of monolithic zirconia crowns.

## Materials and methods

**Figure 1.**
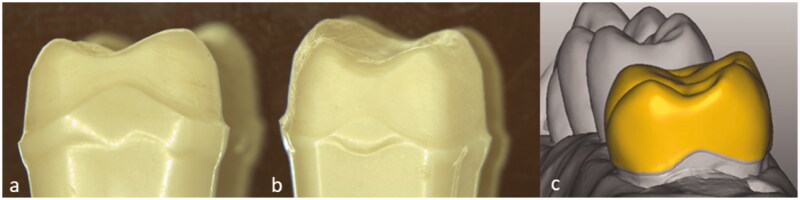
The molar preparation with curvatures on the approximal sides to resemble clinical preparations. (a) mesial view, (b) distal view and (c) the designed crown from the distal view.

A model of a molar tooth (Kavo EWL Model teeth, KaVo Dental, Biberach an der Riss, Germany) was prepared with rounded edges and a shallow chamfer. The model was designed to imitate clinical preparations, with a curvature of the finish line in the mesial and distal areas to allow room for the interproximal gingival papilla. In order to examine the effect of crown axial wall height, the finish line for the mesial wall was prepared with a lower height than the distal wall (Figure 1). The model was scanned digitally (Trios, 3Shape, Copenhagen, Denmark), and a total of 62 monolithic zirconia crowns were made to fit the model, using CAD/CAM technique (Fräsen ver. 4003_0030, ZirkonZahn, Gais, Italy).

Forty 3Y zirconia crowns with 4–6 wt% Y_2_O_3_ and 22 5Y zirconia crowns with ≤12 wt% Y_2_O_3_ were produced according to manufacturer’s instruction to fit the model (Table 1). Due to limited previous data on 5Y-zirconia, the number of specimens was increased from ten to eleven to ensure the statistical power of 80%.

**Table 1.** Material groups used with material names, composition and sintering temperatures, (producers' information).

Groups	Material name	Composition	Sintering conditions
3Y Zirconia	Prettau Zirkon	ZrO_2_ – main component	Heating: 6 °C/min to 1600 °C.
		Y_2_O_3_ – 4–6%	Holding time at max. temp: 120 min
		Al_2_O_3_ – max 1%	Cooling 6 °C/min to room temp.
		SiO_2_, Fe_2_O_3_, Na_2_O < 0.04%	
5Y Zirconia	Prettau Anterior	ZrO_2_ – main component	Heating: 8 °C/min to 1500 °C.
		Y_2_O_3_ – max 12%	Holding time at max. temp: 120 min.
		Al_2_O_3_ – max 1%	Cooling 8 °C/min to room temp.
		SiO_2_, Fe_2_O_3_ < 0.02%	

A stereomicroscope (Leica M205 C, Heerbrugg, Switzerland) was used to examine the crowns at 20× magnification, and all defects and irregularities at the crown margins were registered and graded according to the severity on a scale from 1 to 5, as follows: (1) Smooth edge and no defects, (2) smooth edge and few, small separate defects, (3) several small defects, (4) Rough edge and continuous defects, (5) large defects visible without a microscope [20]. No specimens were excluded.

The crown margins were polished by hand after delivery, according to the protocol and instructions from the manufacturer, using a dental handpiece and diamond-filled rubber wheels with a gradual reduction in grain size (Zirconia polishing kit, Edenta, Switzerland). The crowns were re-examined after polishing, and any alterations in the number or size of defects were recorded.

**Figure 2.**
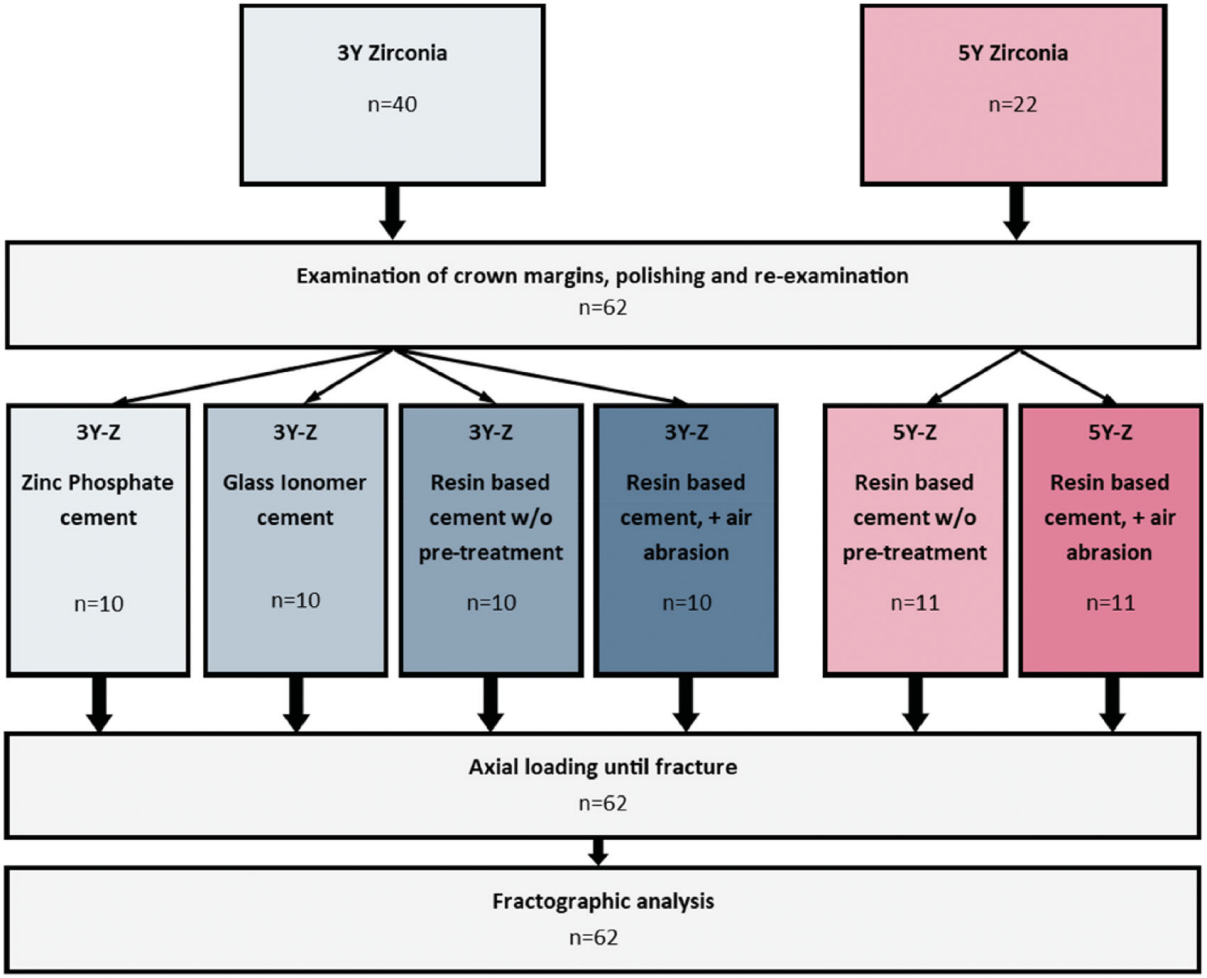
Flow chart of the different cementation procedures.

Impressions of the model were taken with an A-silicone impression material (Affinis, Coltene/Whaledent, Altstätten, Switzerland). The impressions functioned as molds for composite abutment duplicates (SDR^®^ flow+, Dentsply DeTrey GmbH, Konstanz, Germany). The 3Y zirconia crowns were randomly divided into four groups, and were attached to the abutments, using four different cementation techniques; (1) Zinc phosphate cement (Harvard Cement OptiCaps^®^, Harvard Dental International GmbH, Hoppegarten, Germany), (2) Glass ionomer cement (GC Fuji^®^ I CAPSULE, GC Corporation, Tokyo, Japan), (3) Self-adhesive resin-based cement (RelyX™ Unicem, 3 M ESPE, MN, USA), (4) Pre-treatment of the crown with air-abrasion (50 µm alumina particles at 2 bar pressure) and self-adhesive resin-based cement. The 5Y zirconia crowns were divided into two groups and attached to the duplicates with; (3) Self-adhesive resin-based cement, or (4) Air-abrasion pre-treatment and self-adhesive resin-based cement (Figure 2). The abutments were left untreated before cementation

The crowns were attached to the abutment models according to the manufacturer’s instruction for each cement. Excess cement was removed, and after light curing or the appropriate setting time, the crowns were placed in distilled water at 37 °C for 24 ± 1 h.

**Figure 3.**
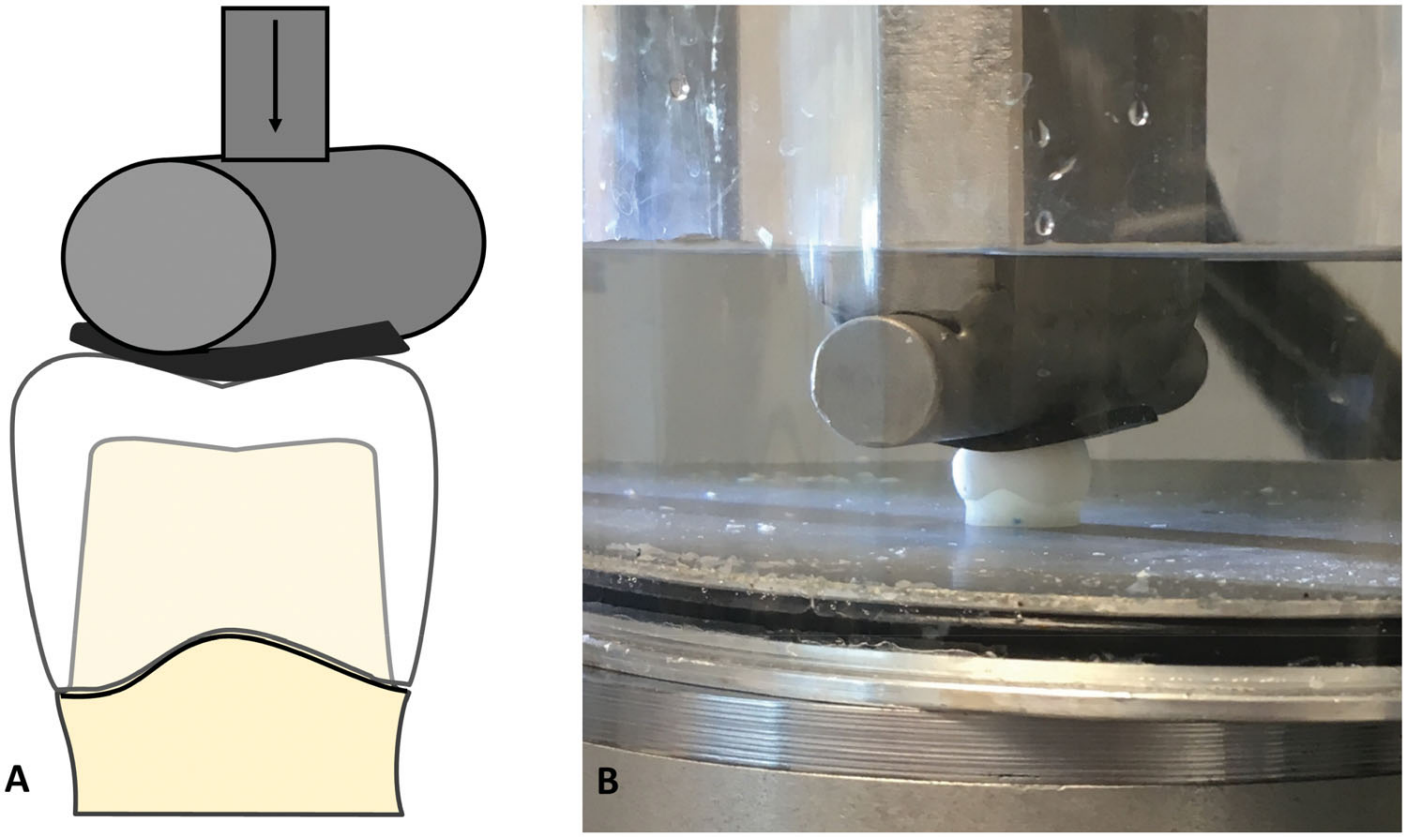
The test set-up. (A) Schematic illustration of the test during loading. (B) Photo of test during loading.

The crowns were subsequently loaded centrally at the occlusal surface with a horizontal steel cylinder of 13 mm in diameter cushioned with a 3 mm thick rubber disc of hardness 90 Shore A (EPDM 90) to avoid contact damages as previously tested (Figure 3) [20–22]. The load was applied in a servo-hydraulic testing system at a rate of 0.5 mm/min until fracture occurred (MTS 852 MiniBionix II, Minnesota, USA). The crowns were submerged in water at room temperature during loading. Load at fracture was recorded and used in the analysis.

The fracture surfaces of all specimens were analyzed by light microscopy using fractographic methods to determine fracture origin and crack propagation. In cases where the fracture origin was difficult to determine, supplementary analyses were performed by scanning electron microscopy (Phenom XL, Endhoven, The Netherlands). At least two samples from each group were analyzed by SEM to verify the light microscopy findings. The inside of the crowns was used to examine the grain structure of the two different materials on two samples from each material group.

Due to a tendency to skewed results non-parametric statistics were used to assess differences among groups with a statistical software package (STATA/SE 16.0). Kruskal–Wallis was used for overall comparison and Mann–Whitney *U*-test was used for between-group comparison. Spearman’s rank correlation coefficient was used to evaluate the association between margin quality and load at fracture. The level of significance was set to .05.

## Results

**Figure 4.**
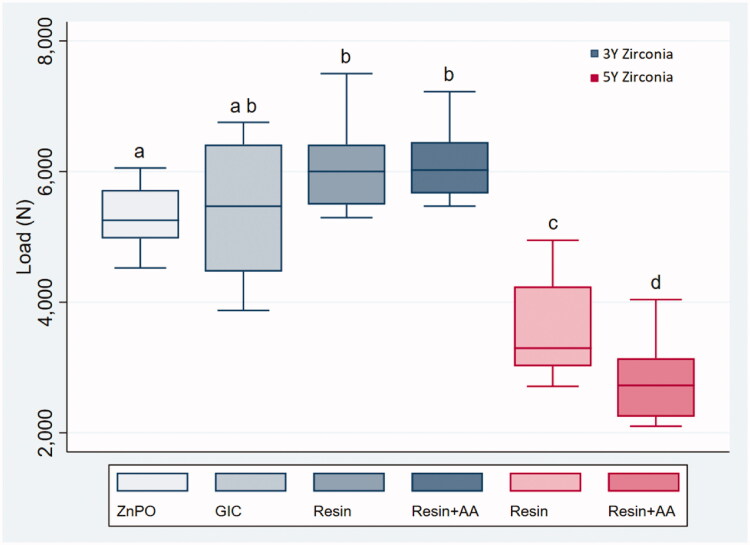
Boxplot of load at fracture (N). Statistically significant differences were found between the two material groups. Statistically significant differences between the different cementation groups are marked with different letters.

There were statistically significant differences between the two material groups (*p* < .001). All crowns fractured during loading, with values ranging from 3873 to 7500 N in the 3Y zirconia crowns, and 2100 to 4948 N in the 5Y zirconia crowns (Figure 4).

Overall comparison of fracture load in the 3Y zirconia groups shows statistically significant differences among the four groups (Kruskal–Wallis test, *p* < .05). The group of crowns attached with zinc phosphate cement fractured at significantly lower loads compared to the resin-based cement groups, with and without air-abrasion, (Mann–Whitney *U*-test, *p* < .012).

In the 5Y zirconia crowns, there was a statistically significant difference between the air-abraded crowns, and the untreated crowns (Mann–Whitney *U*-test *p* < .03).

### Fracture modes

**Figure 5.**
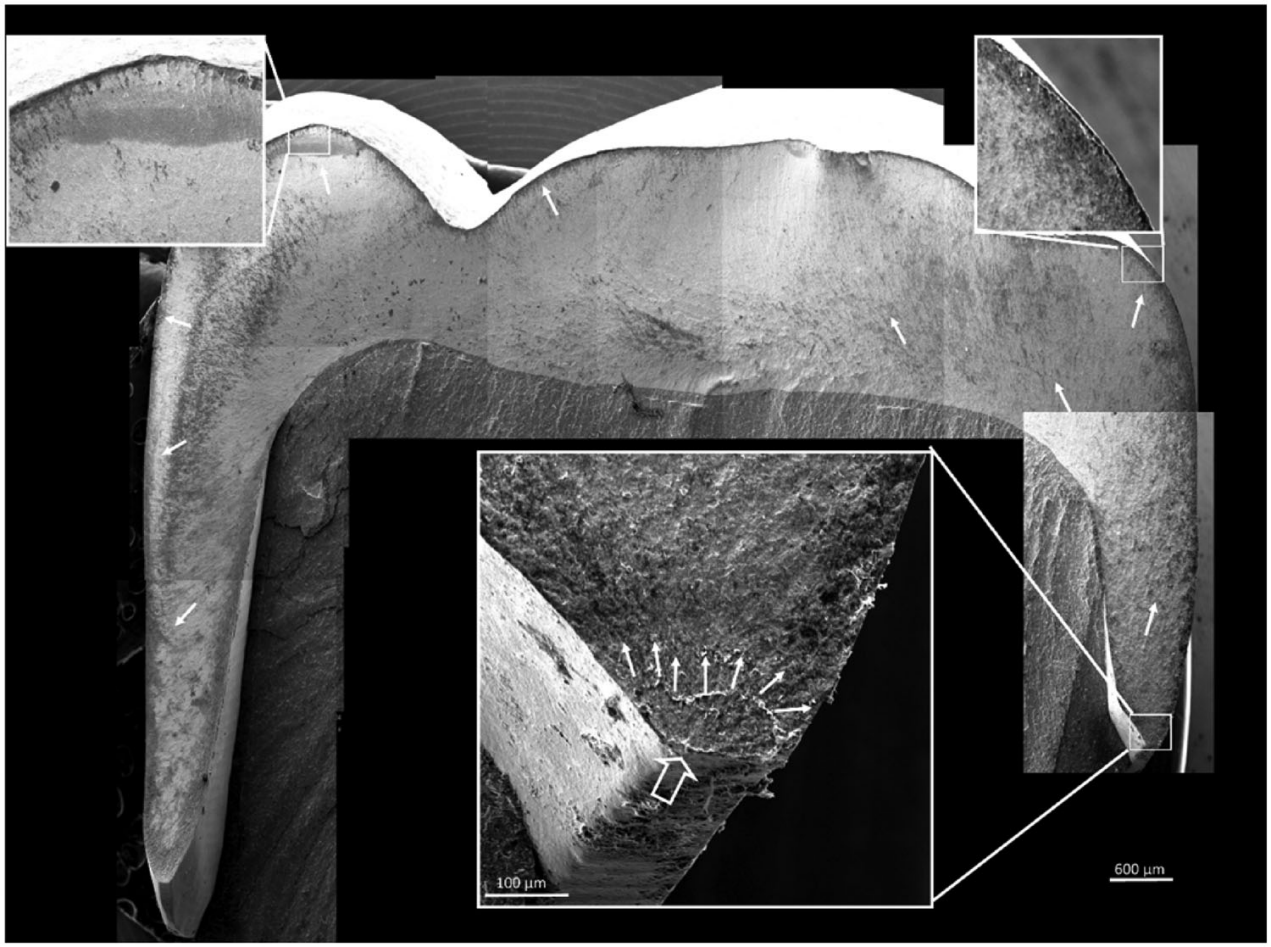
Fractographic map of a fractured crown (3Y zirconia). The origin can be traced back to the crown margin on the mesial curvature (large open arrow). Small white arrows indicate the direction of crack propagation.

No crowns fractured due to contact damage from the indenter. Fracture origin could be identified in all but one crown (Figure 5). The fractographic analyses show that all fractures started in the crown margin’s approximal curvature. The majority (*n* = 51) of the fractures originated in the mesial region, which also is the crown’s shortest wall (Figure 5).

### Margins

There was a statistically significant difference in the margin quality between the two material groups (*p* < .001) The 5Y crowns had generally more frayed margins than the 3Y zirconia crowns, and none of the 5Y crowns were without margin defects. Uneven margins ranged from grade 2 to 5, with a median of 3. The 3Y zirconia crowns had more even margins, where the majority of the crowns had few defects and were categorized as a grade 2.

After polishing, one 3Y crown had to be changed from category 5 to category 2 as the visible defect had become smaller. In the 5Y zirconia group, polishing resulted in four crowns being recategorized due to the crown margin quality worsening: One from category 2–5, one from category 4–5, and two from category 3–4.

There was a statistically significant correlation between the severity of defects in the crown margins and the load at fracture (Spearman’s rho −0.505, *p* < .001). More severe defects gave lower load at fracture. There was no correlation between margin quality and fracture load when the test was sorted by material.

### Microstructure

**Figure 6.**
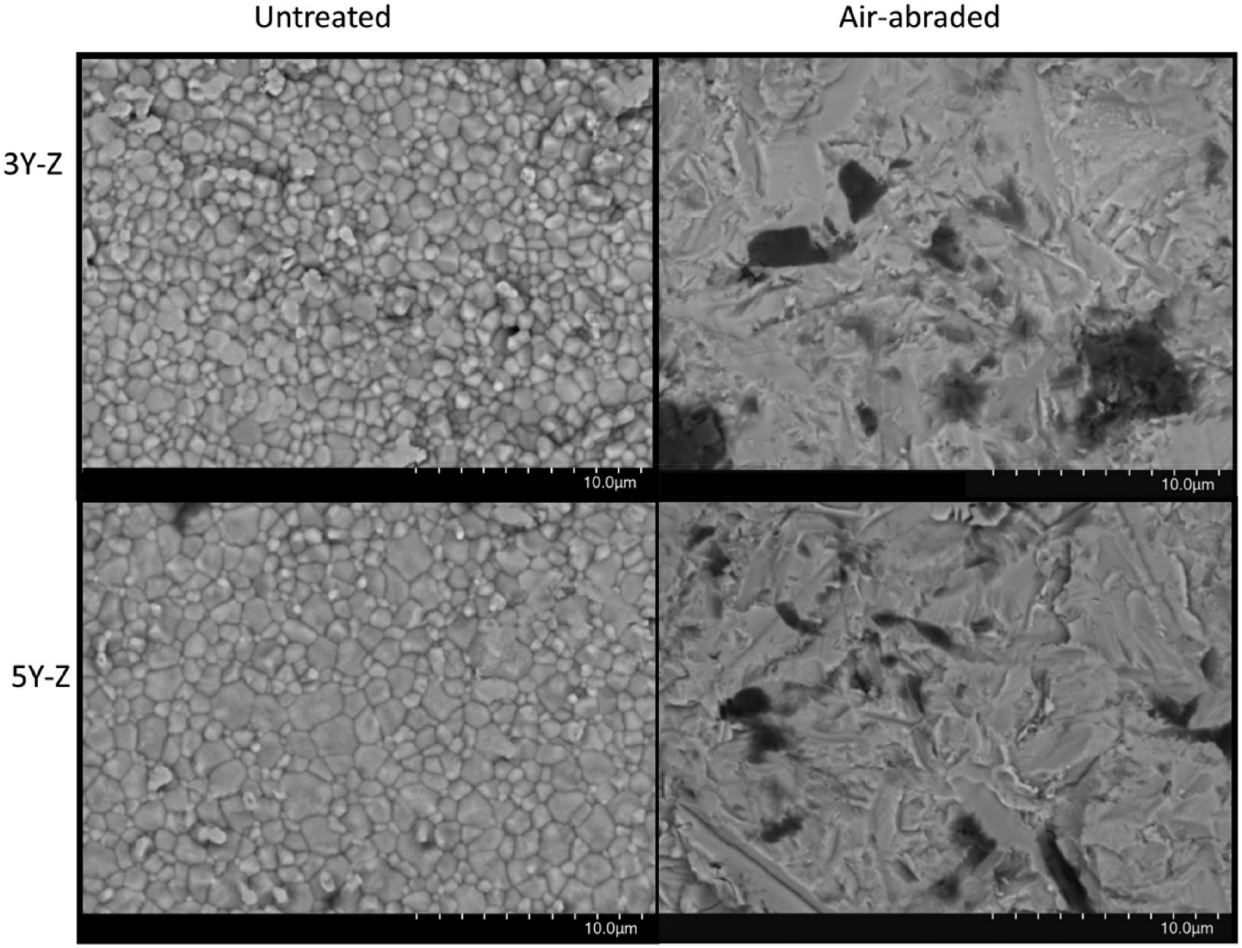
SEM images of the inside of the crowns before (A, C) and after air-abrasion (B, D). The grain structure is clearly different between the 3Y and 5Y zirconia. (A) The 3Y har more homogenous grain structure (1–3 µm). (C) The 5Y has a larger variation in grain size (<1 to >5 µm). (B, D) The air-abrasion results in a severely different surface structure, but there is no apparent difference between the two material surfaces after air-abrasion. Scanning electron microscopy images in back scatter mode.

**Figure 7.**
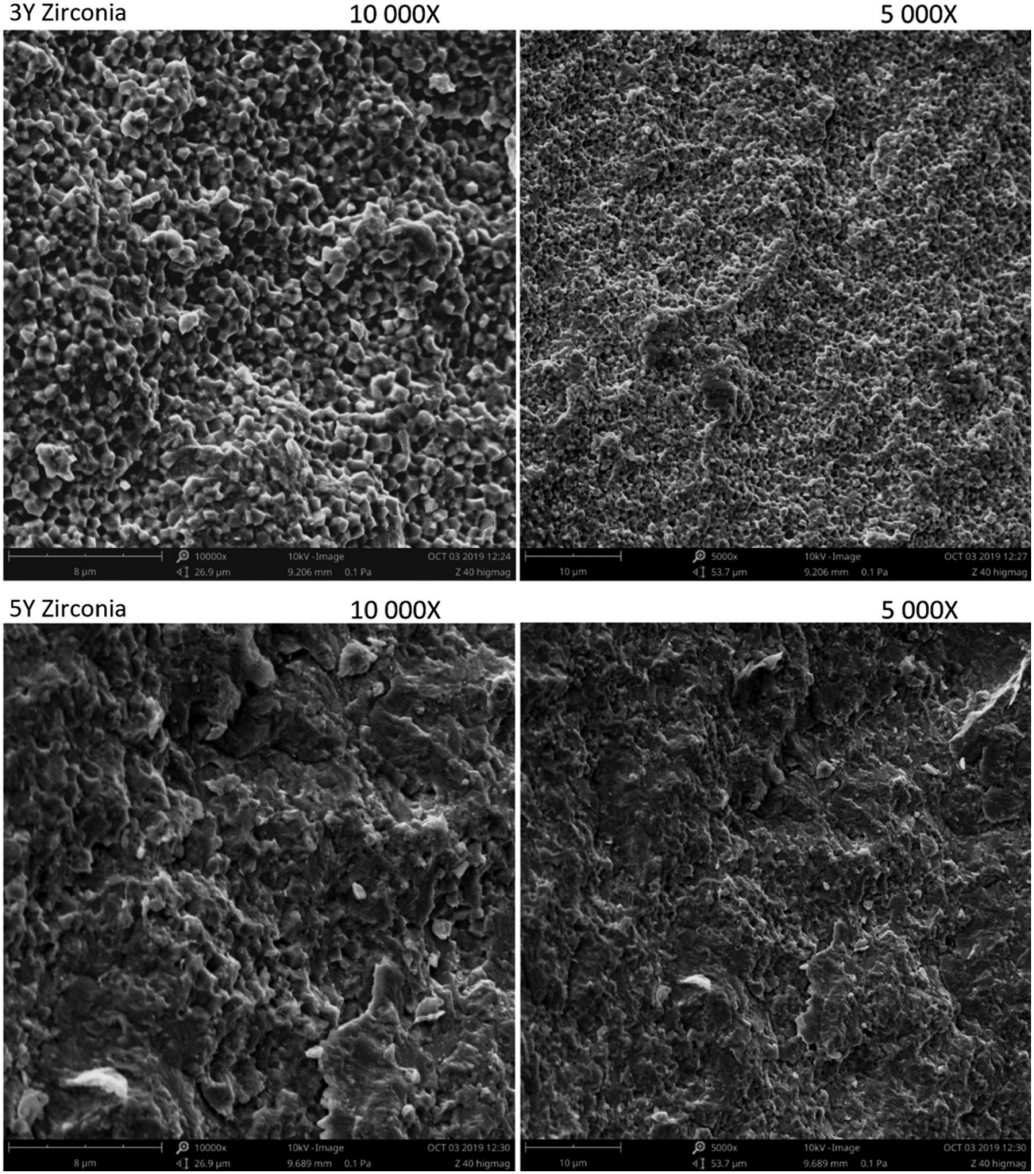
SEM images (secondary emission mode) of typical fracture surfaces of the two material groups. (A, B) The 3Y zirconia display mostly intergranular fracture surface. (C, D) 5Y zirconia display mixed intergranular and transgranular fracture surfaces.

According to the SEM images, the air-abrasion resulted in similar destruction of the surface grain structure in both materials (Figure 6). The fracture surfaces revealed distinct differences in microstructure between the two material groups (Figure 7). The fracture was predominantly intergranular in the 3Y zirconia material compared to mixed transgranular and intergranular fracture in the 5Y zirconia material.

## Discussion

There is a wide selection of cements to choose from, and it is not evident how they affect the clinical success of zirconia crowns. The aim of this study was to evaluate the effect of cement and cementation techniques on the fracture load of monolithic zirconia crowns. The overall results from this study indicate that both cementation technique and the type of cement will affect the fracture load. The null hypothesis was rejected as 3Y zirconia crowns cemented with a resin-based cement fractured at statistically significantly higher loads compared to the crowns cemented with zinc phosphate cement. This suggests that adhesive cement improves crown strength. The present results are not fully in accordance with previous findings as one study finds no effect of cement on the fracture load of zirconia crowns [11], while another shows that resin-based cement do increase the strength [13]. It should however be noted that these studies either did not comment on the fracture modes of the crowns or did not achieve clinically relevant fractures, but rather contact damage from the indenter [21,22].

Leucite-reinforced glass ceramics and feldspathic porcelain crowns achieve significantly higher fracture load when attached with a resin-based cement, compared to conventional cement [23,24]. Silica-based ceramics can achieve a reliable bond to resin by etching with hydrofluoric acid to increase the surface area of the material to allow for greater micromechanical interlocking, and in addition, applying silane to chemically bond the ceramic and resin monomers [25]. These pre-treatments are less efficient on zirconia [26,27], and the material does not bond to resin-based cement as strongly as a silica-based ceramic. Zirconia has however been shown to have significant chemical interactions with phosphate groups that can be found in some resin-based cement [14]. The ability for resin-based cements to bond to zirconia may therefore be of greater importance to the fracture load and strength of zirconia crowns and would explain why the groups cemented with a resin-based cement could withstand the highest loads before fracture.

In addition to an effect on fracture strength, the choice of cement is also important for the retentive properties, as it has been reported that the main mechanical failures in clinical use are loss of retention or veneer material chipping [1]. Because some resin-based cements have chemical interactions with zirconia, the shear bond strength to zirconia is higher, and can therefore be expected to give better retention [28,29]

The group cemented with glass ionomer cement had the largest variation in fracture loads. There was no statistical difference in fracture load to neither the zinc phosphate group nor the resin-based cement groups. The reason for the large scatter is not evident from the present results. All groups fractured at higher values than maximal mastication forces [30], indicating that conventional cement might provide adequate crown strength for clinical use. Resin-based cements are technique sensitive, and susceptible to contamination, making them more demanding to use.

To achieve a desired surface area of the zirconia crowns it is recommended to air-abrade the intaglio surface to improve the micromechanical retention [16]. When examining the surface structure after air-abrasion there were no clear differences between the 5Y zirconia and the 3Y zirconia (Figure 6). Abrading the intaglio surface of the crowns created small defects in the material. These defects did not affect the strength of the 3Y zirconia crowns and can be explained by the stress-induced *t-m* phase transformation that occurs as a result of air abrasion. The phase transformation leads to an increase in surface tension of the material and compressive stresses surrounding the surface flaws [31,32]. This renders the flaws insignificant and has also been shown to increase fracture toughness in some cases [33,34]. It is however important to note that it is uncertain how the phase transformation and aging will affect the mechanical properties and long-term success of the material [35,36]. Because of the higher content of cubic zirconia in the 5Y zirconia crowns, the occurrence of the *t–m* transformation is reduced. This causes the defects in the surface to reduce the strength of the material [32].

Air-abrasion is a common step in the cementation procedure of resin-based cement and is a part of many manufacturers’ instructions when cementing a zirconia crown. It is important to note that they do not differentiate between the different zirconia materials. According to our study, air-abrasion of anterior 5Y zirconia lowers the strength of the crowns and could therefore lead to a higher failure rate in clinical use. Further research is needed to establish recommendations for cementation protocol for 5Y zirconia.

The largest differences in fracture load were found between the 3Y zirconia groups and the 5Y zirconia groups, which was expected due to the differences in strength and fracture toughness in the two materials. The increase of yttria stabilizer leads to a higher percentage of cubic phase in the crystalline structure. While this improves the translucency of the material, it also reduces strength and toughness, as stress-induced phase transformation will be less likely to occur [2,37,38]. The finding that the air-abraded 5Y crowns were weaker than the untreated crowns, supports this assumption.

The margins were inspected before any handling and defects were, therefore, most likely caused by the milling procedure during production. It was found that the 5Y zirconia had more frayed and uneven margins with more severe defects compared to the 3Y zirconia. This demonstrates that the 5Y zirconia is more exposed to damage during manufacturing. The finding that there is a correlation between fracture load and the severity of defects in the crown margins can be explained by the fact that most severe defects were found in the weaker 5Y zirconia material. It can also be argued that these defects might have affected the fracture load, but there was no significant correlation between defects and fracture load when the test was sorted by material. When examining the fracture surface of the two different materials, we found that the 5Y zirconia had a mixed intergranular and transgranular fracture, while the 3Y zirconia crowns primarily had an intergranular fracture surface (Figure 6).

Furthermore, the importance of differentiating between the handling of these two materials becomes apparent when we look at the results from the air abrasion. While the air-abraded 5Y zirconia crowns fractured at significantly lower values than the crowns that had been cemented without pre-treatment, the strength of the pre-treated 3Y zirconia crowns was not affected.

There are several limitations to this study. Standardized *in vitro* testing cannot replicate clinical situations, as clinically observed failures are complex and rely on different variables like tooth anatomy, chewing behavior, oral environment, etc, but well-designed *in vitro* tests can give a better insight into the material *in vivo* [39].

A composite material (SDR flow +) was chosen for the abutment replica, as it has been developed to simulate similar properties to dentine, although with somewhat lower elastic modulus [40]. Human or bovine dentine would perhaps be more clinically relevant regarding the load dispersion, but it would be impossible to achieve a desired standardization of the specimens. It is uncertain how this material will affect the results of the study, as it is a composite material and will possibly have a stronger bond strength to the resin-based cement compared to human dentine. The loads registered in this study are therefore most probably inflated compared to a real-life situation. All crowns fractured at loads well above human mastication loads. This could be explained by the use of pristine specimens, without aging, dynamic loading, or uneven preparation margins. In a clinical situation, crowns would be exposed to temperature fluctuations, as well as consequent lower loads when chewing, which could lead to aging and low-temperature degradation (LTD) [35]. The values obtained in this study are however still useful tools to compare and analyze the differences between the groups. The method used in this study has been shown to give clinically relevant fracture modes, indicating that the forces used *in vitro* manage to simulate the stress situation causing fractures *in vivo* [22,41]. All crowns were examined in a light microscope to determine the fracture pattern, and all fractures originated from the crown margin similarly to the modes described in clinically fractured zirconia crowns [41].

## Conclusion

Within the limitations of this study, it has been shown that resin-based cement improves the fracture load of monolithic zirconia crowns. Pre-treatment with alumina air-abrasion resulted in lower loads at fracture for the 5Y zirconia crowns, while it did not affect the 3Y zirconia crowns.

## References

[1] Sailer I, Makarov NA, Thoma DS, et al. All-ceramic or metal-ceramic tooth-supported fixed dental prostheses (FDPs)? a systematic review of the survival and complication rates. Part I: single crowns (SCs). Dent Mater. 2015;31(6):603–623. 25842099 10.1016/j.dental.2015.02.011

[2] Zhang Y, Lawn BR. Novel zirconia materials in dentistry. J Dent Res. 2018;97(2):140–147. 29035694 10.1177/0022034517737483 PMC5784474

[3] Camposilvan E, Leone R, Gremillard L, et al. Aging resistance, mechanical properties and translucency of different yttria-stabilized zirconia ceramics for monolithic dental crown applications. Dent Mater. 2018;34(6):879–890. 29598882 10.1016/j.dental.2018.03.006

[4] Manicone PF, Rossi Iommetti P, Raffaelli L. An overview of zirconia ceramics: basic properties and clinical applications. J Dent. 2007;35(11):819–826. 17825465 10.1016/j.jdent.2007.07.008

[5] Li RW, Chow TW, Matinlinna JP. Ceramic dental biomaterials and CAD/CAM technology: state of the art. J Prosthodont Res. 2014;58(4):208–216. 25172234 10.1016/j.jpor.2014.07.003

[6] Klimke J, Trunec M, Krell A. Transparent tetragonal yttria-stabilized zirconia ceramics: influence of scattering caused by birefringence. J Am Ceram Soc. 2011;94(6):1850–1858.

[7] Matsuzaki F, Sekine H, Honma S, et al. Translucency and flexural strength of monolithic translucent zirconia and porcelain-layered zirconia. Dent Mater J. 2015;34(6):910–917. 26632242 10.4012/dmj.2015-107

[8] Zhang F, Reveron H, Spies BC, et al. Trade-off between fracture resistance and translucency of zirconia and lithium-disilicate glass ceramics for monolithic restorations. Acta Biomater. 2019;91:24–34. 31034947 10.1016/j.actbio.2019.04.043

[9] Denry I, Kelly JR. State of the art of zirconia for dental applications. Dent Mater. 2008;24(3):299–307. 17659331 10.1016/j.dental.2007.05.007

[10] Pjetursson BE, Sailer I, Zwahlen M, et al. A systematic review of the survival and complication rates of all-ceramic and metal-ceramic reconstructions after an observation period of at least 3 years. Part I: single crowns. Clin Oral Implants Res. 2007;18:73–85. 17594372 10.1111/j.1600-0501.2007.01467.x

[11] Nakamura K, Mouhat M, Nergard JM, et al. Effect of cements on fracture resistance of monolithic zirconia crowns. Acta Biomater Odontol Scand. 2016;2(1):12–19. 27335900 10.3109/23337931.2015.1129908 PMC4894086

[12] Campos F, Valandro LF, Feitosa SA, et al. Adhesive cementation promotes higher fatigue resistance to zirconia crowns. Oper Dent. 2017;42(2):215–224. 27892840 10.2341/16-002-L

[13] Lawson NC, Jurado CA, Huang CT, et al. Effect of surface treatment and cement on fracture load of traditional zirconia (3Y), translucent zirconia (5Y), and lithium disilicate crowns. J Prosthodont. 2019;28(6):659–665. 31145492 10.1111/jopr.13088 PMC6642729

[14] Nagaoka N, Yoshihara K, Feitosa VP, et al. Chemical interaction mechanism of 10-MDP with zirconia. Sci Rep. 2017;7:45563. 28358121 10.1038/srep45563 PMC5372092

[15] Abdalla M, Lung C, Tsoi J, et al. Dental resin-zirconia bonding promotion using high-silica PVD coating with high ionization sputtering processing. Coatings. 2019;9(3):182.

[16] Inokoshi M, De Munck J, Minakuchi S, et al. Meta-analysis of bonding effectiveness to zirconia ceramics. J Dent Res. 2014;93(4):329–334. 24563487 10.1177/0022034514524228

[17] Özcan M, Bernasconi M. Adhesion to zirconia used for dental restorations: a systematic review and meta-analysis. J Adhes Dent. 2015;17(1):7–26. 25646166 10.3290/j.jad.a33525

[18] Blatz MB, Vonderheide M, Conejo J. The effect of resin bonding on long-term success of high-strength ceramics. J Dent Res. 2018;97(2):132–139. 28876966 10.1177/0022034517729134 PMC6429574

[19] Maroulakos G, Thompson GA, Kontogiorgos ED. Effect of cement type on the clinical performance and complications of zirconia and lithium disilicate tooth-supported crowns: a systematic review. Report of the committee on research in fixed prosthodontics of the American Academy of Fixed Prosthodontics. J Prosthet Dent. 2019;121(5):754–765. 30885580 10.1016/j.prosdent.2018.10.011

[20] Schriwer C, Skjold A, Gjerdet NR, et al. Monolithic zirconia dental crowns. Internal fit, margin quality, fracture mode and load at fracture. Dent Mater. 2017;33(9):1012–1020. 28662859 10.1016/j.dental.2017.06.009

[21] Øilo M, Gjerdet NR. Fractographic analyses of all-ceramic crowns: a study of 27 clinically fractured crowns. Dent Mater. 2013;29(6):e78–e84. 23608760 10.1016/j.dental.2013.03.018

[22] Øilo M, Kvam K, Tibballs JE, et al. Clinically relevant fracture testing of all-ceramic crowns. Dent Mater. 2013;29(8):815–823. 23746750 10.1016/j.dental.2013.04.026

[23] Burke FJ. The effect of variations in bonding procedure on fracture resistance of dentin-bonded all-ceramic crowns. Quintessence Int. 1995;26(4):293–300. 7568750

[24] Borges GA, Caldas D, Taskonak B, et al. Fracture loads of all-ceramic crowns under wet and dry fatigue conditions. J Prosthodont. 2009;18(8):649–655. 19682214 10.1111/j.1532-849X.2009.00498.x

[25] Quigley NP, Loo DSS, Choy C, et al. Clinical efficacy of methods for bonding to zirconia: a systematic review. J Prosthet Dent. 2020;125(2):231–240. 32115220 10.1016/j.prosdent.2019.12.017

[26] Borges GA, Sophr AM, de Goes MF, et al. Effect of etching and airborne particle abrasion on the microstructure of different dental ceramics. J Prosthet Dent. 2003;89(5):479–488. 12806326 10.1016/s0022-3913(02)52704-9

[27] Derand P, Derand T. Bond strength of luting cements to zirconium oxide ceramics. Int J Prosthodont. 2000;13(2):131–135. 11203621

[28] Peutzfeldt A, Sahafi A, Flury S. Bonding of restorative materials to dentin with various luting agents. Oper Dent. 2011;36(3):266–273. 21740244 10.2341/10-236-L

[29] Shahin R, Kern M. Effect of air-abrasion on the retention of zirconia ceramic crowns luted with different cements before and after artificial aging. Dent Mater. 2010;26(9):922–928. 20655101 10.1016/j.dental.2010.06.006

[30] Varga S, Spalj S, Lapter Varga M, et al. Maximum voluntary molar bite force in subjects with normal occlusion. Eur J Orthod. 2011;33(4):427–433. 21062965 10.1093/ejo/cjq097

[31] Hannink RHJ, Kelly PM, Muddle BC. Transformation toughening in zirconia-containing ceramics. J Am Ceram Soc. 2004;83(3):461–487.

[32] Yoshida K. Influence of alumina air-abrasion for highly translucent partially stabilized zirconia on flexural strength, surface properties, and bond strength of resin cement. J Appl Oral Sci. 2020;28:e20190371. 32049135 10.1590/1678-7757-2019-0371 PMC6999114

[33] Aurelio IL, Marchionatti AM, Montagner AF, et al. Does air particle abrasion affect the flexural strength and phase transformation of Y-TZP? A systematic review and meta-analysis. Dent Mater. 2016;32(6):827–845. 27083253 10.1016/j.dental.2016.03.021

[34] Kosmac T, Oblak C, Jevnikar P, et al. The effect of surface grinding and sandblasting on flexural strength and reliability of Y-TZP zirconia ceramic. Dent Mater. 1999;15(6):426–433. 10863444 10.1016/s0109-5641(99)00070-6

[35] Lughi V, Sergo V. Low temperature degradation -aging- of zirconia: a critical review of the relevant aspects in dentistry. Dent Mater. 2010;26(8):807–820. 20537701 10.1016/j.dental.2010.04.006

[36] Cattani-Lorente M, Scherrer SS, Ammann P, et al. Low temperature degradation of a Y-TZP dental ceramic. Acta Biomater. 2011;7(2):858–865. 20854937 10.1016/j.actbio.2010.09.020

[37] Zhang F, Inokoshi M, Batuk M, et al. Strength, toughness and aging stability of highly-translucent Y-TZP ceramics for dental restorations. Dent Mater. 2016;32(12):e327–e337. 27697332 10.1016/j.dental.2016.09.025

[38] Pereira GKR, Guilardi LF, Dapieve KS, et al. Mechanical reliability, fatigue strength and survival analysis of new polycrystalline translucent zirconia ceramics for monolithic restorations. J Mech Behav Biomed Mater. 2018;85:57–65. 29857261 10.1016/j.jmbbm.2018.05.029

[39] Kelly JR, Benetti P, Rungruanganunt P, et al. The slippery slope: critical perspectives on in vitro research methodologies. Dent Mater. 2012;28(1):41–51. 22192250 10.1016/j.dental.2011.09.001

[40] Rizzante FAP, Mondelli RFL, Furuse AY, et al. Shrinkage stress and elastic modulus assessment of bulk-fill composites. J Appl Oral Sci. 2019;27:e20180132. 30624465 10.1590/1678-7757-2018-0132 PMC6322642

[41] Øilo M, Hardang AD, Ulsund AH, et al. Fractographic features of glass-ceramic and zirconia-based dental restorations fractured during clinical function. Eur J Oral Sci. 2014;122(3):238–244. 24698173 10.1111/eos.12127 PMC4199274

